# Assessing positive mental health in people with chronic physical health problems: correlations with socio-demographic variables and physical health status

**DOI:** 10.1186/1471-2458-13-928

**Published:** 2013-10-05

**Authors:** Teresa Lluch-Canut, Montserrat Puig-Llobet, Aurelia Sánchez-Ortega, Juan Roldán-Merino, Carmen Ferré-Grau

**Affiliations:** 1 Mental Health Sciences Department, School of Nursing, University of Barcelona, Campus of Health Sciences, Feixa Llarga, s/n, 08907-Hospitalet de Llobregat, Barcelona, Spain; 2 Primary Health Care, Neighbourhood Latino Santa Coloma de Gramanet, Catalan Institute for Health, Barcelona, Spain; 3 Campus Docent, Sant Joan de Déu-Fundació Privada, School of Nursing, University of Barcelona, Barcelona, Spain; 4 CIBERSAM (Centro de Investigación Biomédica en Red de Salud Mental), Sant Boi de Llobregat, Barcelona, Spain; 5 Faculty of Nursing, Rovira i Virgili University of Tarragona, Tarragona, Spain

## Abstract

**Background:**

A holistic perspective on health implies giving careful consideration to the relationship between physical and mental health. In this regard the present study sought to determine the level of Positive Mental Health (PMH) among people with chronic physical health problems, and to examine the relationship between the observed levels of PMH and both physical health status and socio-demographic variables.

**Methods:**

The study was based on the Multifactor Model of Positive Mental Health (Lluch, 1999), which comprises six factors: Personal Satisfaction (F1), Prosocial Attitude (F2), Self-control (F3), Autonomy (F4), Problem-solving and Self-actualization (F5), and Interpersonal Relationship Skills (F6). The sample comprised 259 adults with chronic physical health problems who were recruited through a primary care center in the province of Barcelona (Spain). Positive mental health was assessed by means of the Positive Mental Health Questionnaire (Lluch, 1999).

**Results:**

Levels of PMH differed, either on the global scale or on specific factors, in relation to the following variables: age: global PMH scores decreased with age (r=-0.129; p=0.038); b) gender: men scored higher on F1 (t=2.203; p=0.028) and F4 (t=3.182; p=0.002), while women scored higher on F2 (t -3.086; p=0.002) and F6 (t=-2.744; p=0.007); c) number of health conditions: the fewer the number of health problems the higher the PMH score on F5 (r=-0.146; p=0.019); d) daily medication: polymedication patients had lower PMH scores, both globally and on various factors; e) use of analgesics: occasional use of painkillers was associated with higher PMH scores on F1 (t=-2.811; p=0.006**)**. There were no significant differences in global PMH scores according to the type of chronic health condition. The only significant difference in the analysis by factors was that patients with hypertension obtained lower PMH scores on the factor Autonomy (t=2.165; p=0.032).

**Conclusions:**

Most people with chronic physical health problems have medium or high levels of PMH. The variables that adversely affect PMH are old age, polypharmacy and frequent consumption of analgesics. The type of health problem does not influence the levels of PMH. Much more extensive studies with samples without chronic pathology are now required in order to be able to draw more robust conclusions.

## Background

The notion of Positive Mental Health (PMH) arose out of changes in the general mental health context that took place during the first half of the twentieth century. This period saw a shift in the way that mental disorders were described and approached, namely it became accepted that mental health was more than just the absence of illness, the term “mental health” came to be used as a broad concept covering both mental wellbeing and mental illness, and greater emphasis was placed on the need to work from a community perspective in order to prevent and, especially, to promote mental health. This approach remains a cornerstone of current mental health policy, as is illustrated by the various documents and lines of work being followed by the main political and health institutions [1-8]. In Spain, both the central government and the governments of the various autonomous regions consider the positive aspect of mental health in their mental health plans and strategies [9-11].

Although there is no single definition of mental health, the one published by the World Health Organization in 2001 places considerable emphasis on the positive aspect, it being stated that “*mental health is a state of well-being in which the individual realizes his or her own abilities, can cope with the normal stresses of life, can work productively and fruitfully, and is able to make a contribution to his or her community*” [12]. This definition makes clear that mental health is more than just the absence of illness, and it is this aspect that is captured by the concept of PMH. By moving beyond a narrow vision of disorder it becomes possible to think in terms of promoting mental health by fostering and developing the optimal functioning of the human individual [13,14]. In this same regard the Public Health Agency of Canada (PHAC) states that “*mental health is the capacity of each and all of us to feel, think, and act in ways that enhance our ability to enjoy life and deal with the challenges we face. It is a positive sense of emotional ans spiritual well-being that respects the importance of culture, equity, social justice, interconnections and personal dignity*” [15].

However, this positive perspective on mental health is not founded on a single criterion. Cowen and Kilmer (2002)’s analysis of several studies found a total of 60 criteria or variables referring to the positive side of mental health [16]. Some of them are constructs (such as wellbeing, quality of life, resilience, sense of coherence, optimism, happiness or flourishing) that have generated conceptual and metric approaches of considerable interest [17], with some of the key theoretical approaches being: The conception of Psychological well-being de Bradburn (1969) as the balance between two independent dimensions which he termed positive and negative affect [18]; The salutogenic approach of Antonovsky (1996) which focused on coping rather than stressors and “salutory” factors rather than risk factors [19]; Scheier and Carver (1985) proposed the optimism/pessimism dimension, which they described as a tendency to believe that one will generally experience either good or bad outcomes in life [20]; Character strengths and virtues of Peterson and Seligman [21]; the resilience of Rutter as the capacity to cope with adversity and to avoid breakdown or diverse health problems when confronted with stressors [22]. These theoretical perspectives have also led to the development of important measurement instruments, such as: the Bradburn Affect Balance Scale of Bradburn, [19], The General Well-Being Shedule (GWBS) de Dupuy [23], The Subjective Well-Being de Ryff [24], the Life Orientation Test (LOT) de Scheier and Carver [25], The Warwick-Edinburgh Mental Well-being Scale (WEMWBS) [26].

However, as Lehtinen, Sholman & Kovess-Masfety (2005) points out “*Happiness or life satisfaction are necessarily not the same as positive mental health, although they can be seen as essential components of the construct. More research on the epidemiology of positive mental health is evidently needed*” [27]. Indeed, it is interesting to study the PMH as a construct. Cronbach and Meehl (1955) [28] define a construct as a concept for which there is not a single observable referent, which cannot be directly observed, and for which there exist multiple referents, but none all-inclusive. The question is, however, how many factors or criteria are required to define mental health? The idea of PMH as a construct can be traced back to the pioneering work of Marie Jahoda in 1958 [29], who proposed six general criteria and 16 specific criteria to define the construct PMH. Although Jahoda’s work has been, and continues to be, widely cited, empirical applications of it have yet to be developed. Furthermore, some authors have explicitly stated that their work is based on Jahoda’s proposals but they have not maintained the PMH construct [24,30,31]. The lack of conceptual definitions and specific instruments for evaluating the PMH construct was highlighted by the EUROHIS project, which led to the suggestion that PMH could be assessed by using the Energy and Vitality subscale of the SF-36 [32]. As a result this subscale has been used by recent research on PMH in the European population – Eurobarometers studys [33-35]. Something similar occurred with the MIDUS (Midlife in the United States) [36] in the USA. The aim of this project, which used various assessment scales, was to evaluate health in the midlife population, not simply from the perspective of standard health indicators or in terms of the absence of illness but also in relation to variables such as quality of life and psychological wellbeing. In a similar vein, researchers in Canada have used the definition of mental health formulated by the PHAC (Public Health Agency of Canada) to operationalize the PMH construct into five components: 1) Ability to enjoy life; 2) Dealing with life’s challenges; 3) Emotional well-being; 4) Spiritual well-being; and 5) Social connections and respect for culture, equity, social justice and personal dignity [37,38]. Another recent study of PMH as a construct has been done with Asian population considering the relevance of spiritual and religious practices to mental health [39].

One line of work on PMH is that being developed in Spain, in this case based on the conceptual and metric model of Lluch (1999) [40]. Building on the work of Jahoda, Lluch (1999) proposed a multifactor model of PMH comprising six factors that together constitute the PMH construct: Personal Satisfaction (F1), Prosocial Attitude (F2), Self-control (F3), Autonomy (F4), Problem-solving and Self-actualization (F5), and Interpersonal Relationship Skills (F6). The conceptual description of each factor is shown in Table 1. A measurement instrument, the Positive Mental Health Questionnaire (PMHQ), has also been developed in order to operationalize the model and measure PMH [41].

**Table 1:** Multifactor model of positive mental health (Lluch, 1999)

**PMH factors**	**Definition**
F1: Personal Satisfaction	- Self-concept/Self-esteem
	- Satisfaction with personal life
	- Optimistic outlook on the future
F2: Prosocial Attitude	- Active predisposition towards society
	- Altruistic social attitude; attitude of helping/supporting others
	- Acceptance of others and of differential social characteristics
F3: Self-Control	- Ability to cope with stress/situations of conflict
	- Emotional balance/emotional control
	- Tolerance of frustration, anxiety and stress
F4: Autonomy	- Able to have one’s own standards
	- Independence
	- Self-regulation of one’s behavior
	- Sense of personal security/self-confidence
F5: Problem-Solving and Self-Actualization	- Analytical capacity
	- Able to make decisions
	- Flexibility/able to adapt to change
	- Attitude of continuous growth and personal development
F6: Interpersonal Relationship Skills	- Able to establish interpersonal relationships
	- Empathy/ability to understand the feelings of others
	- Able to give emotional support
	- Ability to establish and maintain close interpersonal relationships.

PMH: Positive Mental Health.

The questionnaire was constructed in three stages. The first was the conceptual definition of the six factors, and the second was the creation of the items and structuring of the questionnaire. We conducted an exhaustive search of questionnaires which measured concepts related to positive mental health or evaluated any of the six factors proposed. The search generated a large pool of items. A measurement instrument was designed with a total of 39 items distributed among the six general factors that made up the hypothetical model. The third stage was validation, in which psychometric reliability and validity analyses were conducted. The internal consistency of the instrument was calculated using Cronbach’s alpha coefficient (1990) [42] and temporal stability was analyzed with the test-retest correlation after 40 days. For content validity, five expert raters were recruited: three psychologists and two psychiatrists specializing in mental health. The raters analyzed the scale applying criteria of relevance and representativeness to assess the fit of the contents of the items to the factors. Other criteria assessed were the length of the questionnaire and the proposed number of factors. The analysis was carried out before the scale was administered. The reports were favourable in all cases, with a unanimous level of agreement. To analyze the criterion validity the General Health Questionnaire, GHQ-12 (Goldberg, 1972) [43] was administered. For construct validity an exploratory factor analysis was performed, using the principal components method and applying oblimin rotation which, according to Nunnally and Bernstein (1995) [44], may provide a more precise factorial definition. We also analyzed the correlations between factors.

The structure and psychometric properties of this instrument are described below in the Methods section. The PMHQ is currently being used by various research groups, both in Spain and internationally [45-47]. A decalogue of practical recommendations has also been designed to show how the theory can be applied in everyday life [48].

The Multifactor Model of Positive Mental Health is based on a holistic view of health and considers that there is a close inter-relationship between physical and mental health. However, the relationship between physical and mental health and between the social, biological and psychological determinants of these positive states is complex. The cooccurrence of physical illness and mental illness is well established and the complex nature of their relationships is being increasingly explored. But research defining positive states of health – mental and physical – is limited. Research has been conducted into the influence of positive states of mind on physical health [49,50], and one of the consequences of this work is that PMH can be regarded as a key aspect of the individual’s overall health status, in relation to which it would act as a protective factor [1,6,7,17,19,22,51]. One sector of the population where it would be important to determine this relationship between physical and mental health is that comprising people with chronic health problems (e.g., hypertension, diabetes or asthma). Therefore, the present study aimed to explore the relationship between PMH and a number of socio-economic variables and physical health conditions in a sample of people with chronic diseases of this kind.

## Methods

A cross-sectional, descriptive and correlational study was carried out between January and May 2012. The study sample comprised people with chronic physical health diseases who were being seen by a nurse attached to a primary care center in Barcelona (Spain). As is common in Spain this primary care center was organized into what are known as “basic healthcare units”, each one comprising a general practitioner and associated staff. A total of 1295 adults were registered with the Basic Healthcare Unit (BHU) studied here. All patients aged over 45 who were being seen by the nurse of this basic healthcare unit were eligible for inclusion, although participation was strictly voluntary. Patients were excluded if they were not registered with the basic healthcare unit in question, or if they declined to take part. A total of 441 people aged over 45 were registered with the said healthcare unit. The sample was chosen by means of accidental (non-probability) sampling, yielding a sample size of n = 259 with an α risk = 0.05 for a precision of 5%.

The study variables were grouped into four blocks: a) socio-demographic variables: age (45–55; 56–65; 66–75; y >75 years), gender (male/female), level of education (none, primary, professional training, high school), marital status (single, married, widowed, divorced, civil partner), number of children, nationality/place of birth (country and autonomous region within Spain); b) employment variables: profession (housewife, industrial worker, service sector, construction) and employment status (permanent, temporary, unemployed, retired); c) physical health variables: type of chronic condition according to the International Classification of Diseases (ICD-9), number of chronic conditions, prescribed drug consumption (0 to 5 different drugs per day, 6 or more different drugs per day) and frequency of analgesic use (occasionally/daily); and d) the PMH variable, assessed according to the six factors of the above mentioned Multifactor Model of Positive Mental Health: Personal Satisfaction (F1), Prosocial Attitude (F2), Self-control (F3), Autonomy (F4), Problem-solving and Self-actualization (F5), and Interpersonal Relationship Skills (F6). PMH was also evaluated as a single variable based on the global score for the six factors.

The socio-demographic and employment variables, as well as those related to physical health conditions, were assessed by means of an ad hoc instrument constructed with different types of closed question, depending on whether the variables were dichotomous or multiple.

The PMH variable was assessed using the Positive Mental Health Questionnaire (PMHQ; Lluch, 1999) [40,41]. This questionnaire comprises 39 items which are unevenly distributed across the six factors that define the construct: F1-Personal Satisfaction (8 items), F2-Prosocial Attitude (5 items), F3-Self-control (5 items), F4-Autonomy (5 items), F5-Problem-solving and Self-actualization (9 items), and F6-Interpersonal Relationship Skills (7 items). The items take the form of positive or negative statements which are responded to a scale ranging from 1 to 4, according to how frequently they occur: always or almost always, quite often, sometimes, never or rarely. The questionnaire provides a global score for PMH (sum of the item scores) as well as specific scores for each factor. The global PMH value ranges from 39 points (low PMH) to 156 points (high PMH). The minimum and maximum values for each factor are: 8–32 (factor F1), 5–20 (factor F2), 5–20 (factor F3), 5–20 (factor F4), 9–36 (factor F5) and 7–28 (factor F6).

The PMHQ has been validated in a population of students and in the general population. The first study was conducted with a sample of 387 first- and second-year students at the School of Nursing at the University of Barcelona, during the 1998–1999 academic year. To assess the test-retest reliability, the questionnaire was administered after an interval of 40 days to 298 subjects. All questionnaire items obtained discrimination scores above 0.25. In the exploratory factor analysis the six factors extracted accounted for 46.8% of the total variance of the questionnaire. In the resulting factor matrix, the weights of each item with respect to the factor extracted were above 0.40 in all cases. The overall correlation of the GHQ-12 with the PMHQ was r = −0.41. The Cronbach's alpha (internal consistency) of the global scale was 0.91, and by factors the values obtained were: F1 = 0.83; F2 = 0.58; F3 = 0.81; F4 = 0.77; F5 = 0.79; and F6 = 0.71. In the test re-test analysis the values were similar: global PMH =0.85; F1 = 0.79; F2 = 0.60; F3 = 0.72; F4 = 0.77; F5 = 0.77; F6 = 0.72. The second validation study in the general population was conducted with a sample of 581 subjects, in 2006. For the criterion validity of the WHOQOL-BREF was added. In exploratory factor analysis the six factors extracted accounted for 44.6% of the total variance of the questionnaire. The overall correlation of the PMHQ with the GHQ-12 was r = −0.43 and with the WHOQOL-BREF r = 0.54 [52]. The Cronbach's alpha (internal consistency) of the overall scale was 0.88 (men = 0.87 and women = 0.89) and according to factors the values obtained were: F1 = 0.77; F2 = 0.59; F3 = 0.78; F4 = 0.72; F5 = 0.75; and F6 = 0.66. In the retest analysis (with an interval of 35 days) similar values were obtained: global PMH =0.84; F1 = 0.77; F2 = 0.55; F3 = 0.74; F4 = 0.82; F5 = 0.68; F6 = 0.60.

The nurse attached to the basic healthcare unit gave out the PMHQ to patients, who completed it alone once consent had been obtained from both the primary care center and the patients themselves.

Data were analyzed using the software package PASW 18. In general, tests were considered significant when they had a *p* value less than 0.05 (alpha significance level = 5). All tests were two-tailed.

Categorical variables were analyzed in the form of frequency and percentage tables, while numerical variables were considered in terms of basic descriptive statistics (mean, median, quartiles, standard deviation, and standard error). Means of a normally distributed numerical variable were compared using the Student’s *t* test when there were two groups, and by one-way ANOVA when there were more than two groups. The non-parametric Mann–Whitney U test was used for groups with a small sample size.

Two numerical variables were compared by means of Pearson or Spearman correlations (depending on whether or not the two variables were normally distributed).

We also analysed the psychometric properties of reliability and validity of the PMHQ. The internal consistency of the instrument was calculated using the Cronbach alpha coefficient (1990) [42] and a principal components factor analysis was performed using oblimin rotation.

This study was approved by the Ethical Committee of Clinical Research of the *Fundació* Jordi *Gol i Gorina* (Primary Care Research Institute, Catalonia, Spain).

## Results

### Descriptive socio-demographic data

There was a slightly higher proportion of women than men (54.1% versus 45.9%). The most common age of patients was between 66 and 75 years (43.2%). The majority of them (81.9%) were married, and the most common number of children was two (54.1%). A high proportion of the sample had no formal schooling (42.1%) or had only completed primary education (49%). The large majority (90%) had been born in a different autonomous region of Spain to the one in which they currently lived (Catalonia), and were therefore immigrants to this region. As regards their employment status, 67.9% were retired, 16.6% had a permanent job, 12.3% were unemployed and 3.1% had no stable employment. By profession the sample could be classified as follows: 33.9% were housewives, 32.4% worked in the service sector, 8.5% worked in industry and 7.3% were employed in the construction sector.

### Descriptive physical health conditions data

The most frequent chronic health condition was hypertension (68,7%), followed by Hypercholesterolemia (55.2%), Diabetes Mellitus (26.3%) and Osteoarthritis (17.4%). Sixty per cent of the sample had one or two health conditions, while 35.5% had three or more. The majority of patients (69.1%) were taking between one and five different prescribed drugs per day, with the remaining 30.9% receiving polymedication (6 or more different drugs per day). As regards consumption of analgesics, 68.7% did not take painkillers or only did so occasionally, while the remaining 31.3% of patients used them on a daily basis.

### Psychometric properties of the PMHQ

In the principal components factor analysis the six factors extracted explained 48.3% of the total variance of the questionnaire. The F1 factor explained the highest percentage of explained variance (21.5%) while the other five factors participated to a much lower extent (F2 = 9.2%; F3 = 5.1%; F4 = 4.7%; F5 = 4.1; F6 = 3.6). In the resulting factor matrix, the weights of each item with regard to the factor extracted were greater than 0.40 in all cases.

The Cronbach's alpha (internal consistency) of the overall scale was 0.91 and for the factors the values obtained were: F1 = 0.75; F2 = 0.60; F3 = 0.76; F4 = 0.64; F5 = 0.82; and F6 = 0.64.

### Descriptive positive mental health and age groups data

Results from the PMHQ showed that the mean global PMH score was X = 118 (SD = 15.5). The global PMH score decreased with age. The mean score among patients aged 45–55 years was X = 121.29 (SD = 16.70), while in those aged 76 and over it was X = 113.88 (SD = 15.85), which corresponded to the lowest level of PMH in this sample. PMH scores were practically the same in the 56–65 and 66–75 age groups (Table 2).

**Table 2:** Levels of PMH score, both global and by factor according to age

**Age for intervals**	**Mean (SD)**	**Min**	**Max**
**Global PMH**			
45-55	121.2 (7,16)	87	150
56-65	119.3 (3,14)	86	15
66-75	119.0 (7,15)	83	153
>76	113.8 (7,15)	82	151
**Fac1** Personal Satisfaction			
45-55	24.8 (3,5)	8	31
56-65	25.2 (3,8)	15	132
66-75	26.9 (3.8)	16	32
>76	25.8 (3.7)	17	32
**Fac2** Prosocial Attitude			
45-55	16.5 (2.4)	12	20
56-65	16.7 (2.2)	10	20
66-75	16.3 (2.4)	10	20
>76	16.3 (2.8)	9	20
**Fac3** Self Control			
45-55	14.0 (4.0)	6	20
56-65	13.9 (3.1)	7	20
66-75	14.0 (3.3)	5	20
>76	12.9 (3.0)	6	20
**Fac4** Autonomy			
45-55	15.8 (2.2)	6	20
56-65	15.6 (3.0)	8	20
66-75	15.4 (2.7)	6	20
>76	15.9 (2.1)	11	20
**Fac5** Problem-Solving and Self-Actualization			
45-55	28.2 (5.1)	19	36
56-65	26.9 (4.7)	18	36
66-75	26.4 (5.3)	15	36
>76	22.9 (5.6)	12	35
**Fac6** Interpersonal Relationship Skills			
45-55	21.6 (3.2)	15	27
56-65	20.7 (3.7)	10	28
66-75	20.7 (3.5)	11	28
>76	19.9 (3.5)	10	28

PMH: Positive Mental Health.

Minim: Minimum.

Maxim: Maximum.

SD: Standard Deviation.

The analysis of PMH levels by age and by specific factor revealed the following: 1) the highest level of Personal Satisfaction (F1) was reported by patients aged 65–75 years, while the lowest level corresponded to the 45–55 age group; 2) scores for Prosocial Attitude (F2) were very similar across all the age groups; 3) the lowest scores on Self-control (F3) were obtained by patients aged 76 and over, while the highest scores corresponded to the 45–55 and 65–75 age groups; 4) scores for Autonomy (F4) were very similar across all the age groups; 5) Problem-solving ability and Self-Actualization (F5) decreased with age; and 6) Interpersonal Relationship Skills (F6) decreased with age.

Global scores on the PMHQ were then analyzed according to three levels of mental health: low, moderate and high. A low level was defined as more than one standard deviation below the mean obtained in the study sample (X = 118.6), a moderate level corresponded to one standard deviation (SD = 15.5) either side of the mean, and a high level was equivalent to more than one standard deviation above the mean. This categorization revealed that 16.9% of the sample had a low level of mental health, 65.5% a moderate level and 17.6% a high level.

### Correlations between PMH scores and socio-demographic characteristics

Analysis of PMH scores in relation to gender (Table 3) revealed significant differences on four factors. On F1 (Personal Satisfaction) the mean score for men was higher than that of women (t = 2.203; p = 0.028). Conversely, on F2 (Prosocial Attitude) women obtained a higher mean score than did men (t = −3.086; p = 0.002). On F4 (Autonomy) the mean score for men was higher than that of women (t = 3.182; p = 0.002). And, on F6 (Interpersonal Relationship Skills) women obtained a higher mean score than did men (t = −2.744; p = 0.007).

**Table 3:** Relationship between PMH and gender

**PMH**	**Male** **Mean (SD)** **n = 119**	**Female** **Mean (SD)** **n = 140**	**t**	p*
**Global PMH**	118.5 (15.8)	118.5 (3,15)	.027	0.978
**Fac1.** personal satisfaction	26.2 (3.6)	25.1 (4.2)	2.203	0.028
**Fac2.** prosocial attitude	15.9 (2.3)	16.9 (2.4)	−3.086	0.002
**Fac3.** self control	13.7 (3.3)	13.8 (3.3)	-.271	0.786
**Fac4.** autonomy	16.2 (2.5)	15.1 2.7	3.182	0.002
**Fac5.** problem-solving and self actualization	26.3 (5.6)	26.1 (5.1)	.230	0.818
**Fac6.** interpersonal relationship skills	20.0 (3.5)	21.2 (3.4)	2.744	0.007

PMH: Positive Mental Health.

SD: Standard Deviation.

*Student’s *t* test.

As regards levels of PMH according to age, Pearson’s r showed a significant correlation (Table 4) between age and: a) the global score: as age increased, the global level of PMH tended to fall (r = −0.129; p = 0.038); and b) Problem-solving and Self-actualization (F5): the level of PMH on this factor tended to decrease with age (r = −0,269; p = 0.001).

**Table 4:** Relationship between PMH and age (n = 259)

**PMH**	**r**	p*
**Global PMH**	-.129	0.038
**Fac1.** personal satisfaction	.072	0.251
**Fac2.** prosocial attitude	-.048	0.439
**Fac3.** self control	-.067	0.280
**Fac4.** autonomy	-.034	0.591
**Fac5.** problem-solving and self actualization	-.269	0.000
**Fac6.** interpersonal relationship skills	-.115	0.066

PMH: Positive Mental Health.

*Pearson correlation coefficient.

With respect to marital status there were no differences in the level of PMH between any of the sub-categories of this variable, neither for those patients with a partner or for those who lived alone (regardless of whether they were single, widowed or divorced). Neither were any significant differences observed in the level of PMH according to the number of children a patient had.

The relationship between PMH scores (both global and by factor) and educational level was analyzed by means of Spearman correlations. Although none of the correlations was statistically significant, a strong trend towards significance was observed for the global PMH score (r = 0.115; p = 0.064) and for the scores on F4-Autonomy (r = 0.114; p = 0.068) and F5-Problem-solving and Self-actualization (r = 0.123; p = 0.048). For both the global score and the score on these two factors the analysis showed that the level of PMH tended to increase with higher levels of education.

The one-way ANOVA, carried out to examine the relationship between level of PMH and place of birth, revealed no significant relationship. Neither was there any significant difference with respect to type of job or current employment status.

### Correlations between PMH scores and physical health conditions

For each of the 16 chronic health diseases detected we compared the PMH scores (both global and by factor) obtained by patients with and without the condition (Table 5). Basic descriptive statistics (frequency, mean, standard deviation and standard error) were calculated for PMH levels in the two groups (i.e., those with and without each health condition). The Student’s *t* test was used to compare the means between these two groups.

**Table 5:** Relationship between PMH and physical health condition (Hypertension)

**PMH**	**Yes** **Mean (SD)** **n = 178**	**No** **Mean (SD)** **n = 81**	**t**	p*	d^**^
**Global PMH**	117.8 (15.7)	120.0 **(**15.0)	1.042	0.298	0.14
**Fac1.** personal satisfaction	25.7 (4.1)	25.4 (3.6)	0.698	0.486	0.09
**Fac2.** prosocial attitude	16.4 (2.4)	16.6 (2.3)	0.756	0.433	0.10
**Fac3.** self control	13.8 (3.2)	13.9 (3.4)	0.260	0.795	0.03
**Fac4.** autonomy	15.4 (2.9)	16.1 (2.2)	2.165	0.032	0.29
**Fac5.** problem-solving and self actualization	25.9 (5.2)	26.8 (5.5)	1.955	0.282	0.26
**Fac6c** interpersonal relationship skills	20.5 (3.5)	21.1 (3.4)	1.336	0.183	0.18

PMH: Positive Mental Health.

SD: Standard Deviation.

*Student’s *t* test.

^**^Standardized Mean Difference.

Significant differences in the level of PMH were only found in relation to hypertension and on F4 (Autonomy). Specifically, the mean PMH score obtained on this factor by patients without hypertension was higher than that of those with this condition (t = 2.165; p = 0.032). In general, the magnitude of effect sizes is small. But, as Cohen (1988) [53] pointed out, small differences may be of interest in new areas of research or in studies with modest designs.

No significant differences were found in relation to the health conditions Diabetes Mellitus, Hypercholesterolemia, Osteoarthritis, Osteopathy, Hypothyroidism and Cancer. With regard to Heart Failure, Asthma, Arthritis, Hepatitis, Psoriasis, Alzheimer, Parkinson, Obesity and Fibromyalgia the number of patients was too small to allow an analysis of correlations.

As regards the amount of medication that patients were consuming daily there were significant differences in relation to the global PMH score and the score on F3 (Self-control), F4 (Autonomy) and F5 (Problem-solving and Self-actualization). Specifically, the median score of polymedication patients was significantly lower as compared with patients taking fewer than six prescribed drugs daily for: global PMH (t = 2.423; p = 0.017); F3 (t = 2.552; p = 0.012), F4 (t = 2.100; p = 0.038); and F5 (t = 2.617; p = 0.010).

With respect to analgesic consumption, whether or not a patient took painkillers had no effect on PMH scores, neither globally nor by factor. We then analyzed PMH levels among those patients who did use analgesics (N = 181) according to how often they did so (daily or occasionally). A significant difference was only observed in relation to F1 (Personal Satisfaction), where the mean score of patients who occasionally took painkillers was significantly higher than that of those who used them on a daily basis (t = −2.811; p = 0.006).

Finally, the relationship between PMH levels and the number of chronic health conditions was examined by means of Spearman’s correlation coefficients. The analysis only revealed a significant correlation in relation to F5-Problem-solving and Self-actualization (r = −0.146; p = 0.019), such that PMH scores on this factor were lower when the number of health conditions was higher. For the global PMH score the correlation was close to but failed to reach statistical significance (r = −0.019; p = 0.081).

## Discussion

### Perceived PMH

Most of the patients in this sample presented moderate or high levels of PMH. This finding is consistent with data from the Eurobarometer survey 2010 [35] which have found that people generally feel more positive than negative, in other words, they are able to experience positive emotions more often than negative ones. However, there has been a decrease in relation to positive feelings since the Eurobarometer survey conducted in 2006, with fewer people reporting that they have experienced positive emotions all or most of the time over the last month [34]. It would be interesting to carry out longitudinal studies in this regard.

The present results show that age is related to PMH, since global PMH scores were significantly lower among the oldest group of patients (aged 76 and over). This is consistent with previous findings [34,37]. However, it contrasts with the results of the MIDUS study [54], in which the best levels of PMH were reported by people aged 65–74 years. Our results for age can be explained by considering the different factors that make up the Multifactor Model of Positive Mental Health on which this study is based. Specifically, the finding that the global PMH score decreased with age is consistent with the fact that three of the model’s six factors showed a relationship to age: older age was associated with lower scores on Self-control (F3), Problem-solving and Self-actualization (F5), and Interpersonal Relationship Skills (F6). In fact, on none of the factors did the oldest age group score higher than the other groups. This suggests that after the age of 75, perceived positive mental health decreases on certain factors (F3, F5 and F6) and is maintained on others (F1, F2 and F4). It should also be noted that the youngest age group (45–55 years) obtained the highest scores on the global PMH scale and on two factors (F5 and F6). By contrast, the same group was associated with the lowest PMH scores on Personal Satisfaction (F1). More detailed studies are now required to explore these findings in greater depth.

As regards the relationship between PMH and gender there were no differences between the global scores of men and women. However, the analysis by factors showed that women scored higher on F2 (Prosocial Attitude) and F6 (Interpersonal Relationship Skills), while men scored higher on F1 (Personal Satisfaction) and F3 (Self-control). These results are consistent with the Canadian study [34], in which men had higher levels of PMH in relation to emotional coping skills and wellbeing, while women scored higher on spiritual values and social relationships. In the MIDUS study [54] women reported lower levels of PMH.

We observed no differences in PMH scores between employed and unemployed patients, a finding that contrasts with those studies which have reported lower levels of mental health among the unemployed and those with unstable jobs [37]. This aspect requires further exploration in future studies.

Overall, educational level showed no significant correlation with perceived PMH, although a strong trend towards significance was observed for the global score and for the scores on F4 (Autonomy) and F5 (Problem-solving and Self-actualization). While acknowledging that larger-scale studies are need to confirm these findings the results are nonetheless in line with those obtained in the Canadian study [37], in which the highest levels of coping skills were shown by those with university-level education. Conversely, in the MIDUS study [54] education was not related to the perceived level of PMH.

According to the present results, living alone or with a partner did not have a significant effect on the level of PMH. This contrasts with the findings of previous research, such as the Eurobarometer 2010 [35], where living alone was associated with greater consumption of antidepressants, or the Canadian study [37], which found that people who lived alone or who didn’t have a partner were more likely to report lower levels of emotional wellbeing. It should be noted, however, that what is normally assessed is the level of social support, rather than simply living alone or with someone else. Our study did not take this aspect into account, and it would be interesting to explore this variable in future research.

### PMH and physical health conditions

Most of our subjects had hypertension and hypercholesterolemia or diabetes. The association of two or more chronic conditions was very common, and the proportion of different conditions corresponds to the statistical data published by public health agencies and organizations [55,56]. This was also the case for daily medication consumption: 30.9% of the sample took more than six different prescribed drugs per day, and 31.3% took painkillers every day).

Overall, there was no chronic health condition that produced significant differences in the level of PMH reported by patients with and without the condition, which suggests that having a particular chronic health problem does not in itself influence the degree of PMH. Our research is entirely exploratory and the sample size is very small. Further studies will be needed to draw more robust conclusions. While some studies have found a relationship between physical health and mental health problems [57], our study does not allow us to draw conclusions in this regard. In order to examine whether having a chronic disease is associated with PMH it would be necessary to perform this same study with a similar sample of subjects from the general population who do not suffer any chronic disease.

One chronic health condition, hypertension, yielded a significant difference on one of the factors of PMH. Specifically, patients without hypertension scored higher on F4 (Autonomy) than those with this condition. However, this result should be considered with caution because the magnitude of the effect size is small. For the remaining chronic problems the sample size was too small to allow conclusions to be drawn, hence the need for larger-scale studies in the future. This is an aspect worth exploring since the literature supports the relationship between perceived health status and quality of life, personal satisfaction and PMH [38,58,59]. For example, the MIDUS study [54] found that for persons aged 25–75 years those with physical health diseases reported lower levels of PMH than did those without such diseases. Similarly, people surveyed in the Eurobarometer study 2010 [35] also stated that physical health diseases had a negative impact on their PMH.

In this context, an important finding of the present study is the relationship between PMH and the amount of daily medication. Patients who took more medication reported lower levels of PMH, both in terms of the global score and on three of the six factors (Self-control, Autonomy, and Problem-solving and Self-actualization). However, the specific analysis for analgesic consumption showed that this was only associated with differences on F1 (Personal Satisfaction): patients who did not take painkillers on a daily basis reported greater satisfaction than those who did.

Some authors state that people with low levels of emotional stability and whose response to situations is often disproportionate or accompanied by irritability and anxiety may be more likely to develop a mental disorder in the future [60,61]. This suggests that positive mental health could be a key factor in terms of increasing the level of general health, and also for detecting those most at risk of developing a mental disorder [62,63].

### Limitations

One of the main limitations of this study is the sample size, such that the results need to be verified by studying much larger groups of people with chronic health diseases. This is especially the case for those health conditions where 1) it was not possible to examine the correlations due to the small number of patients (e.g., heart failure, asthma, etc.), 2) the results show a trend towards a significant relationship (e.g., obesity and Alzheimer’s disease), and 3) those conditions, such as hypertension, for which a significant relationship was observed but where a larger sample would be needed to generalize the results and draw firmer conclusions.

A further limitation is that all the subjects had at least one chronic health disease, and it would be necessary in the future to study people without any such condition in order to determine whether the same pattern of results is obtained.

## Conclusions

The study sample comprised older adults (aged 45 and over) whose socio-demographic profile and physical health status corresponded to the general population of people with chronic health problems who are seen in the primary care setting. In this context the main role of the nursing team is to monitor and manage the physical health problem.

The four most prevalent chronic health diseases in this sample were hypertension, hypercholesterolemia, diabetes and osteoarthritis. Overall, there was no chronic health condition that produced significant differences in the level of PMH reported by patients with and without the condition. This suggests that having a particular chronic health problem (as opposed to any other one) does not in itself influence the degree of PMH. However, there was one chronic health disease, namely hypertension, which did produce a significant difference on one of the PMH factors: patients without hypertension scored higher on F4 (Autonomy) than did those with this condition, but the magnitude of the effect size was small.

As regards medication, 30.9% of the sample took more than six different prescribed drugs per day (polymedication) and 31.3% used some form of analgesic on a daily basis. PMH showed a negative association with the amount of daily medication: the more medication that was taken the lower the reported level of PMH, both globally and on various factors. Patients who took painkillers every day scored higher on F1 (Personal Satisfaction) than did those who made less frequent use of analgesics.

Reported levels of PMH decreased with age, both in terms of the global score and the scores on F3 (Self-control), F5 (Problem-solving and Self-actualization) and F6 (Interpersonal Relationship Skills). Globally, there were no significant differences between the PMH of men and women, although the analysis by factors showed that women scored higher on F2 (Prosocial Attitude) and F6 (Interpersonal Relationship Skills), while men scored higher on F1 (Personal Satisfaction) and F3 (Self-control).

In summary, the specific type of chronic physical health problem suffered do not appear to influence the level of positive mental health. This is an important finding in that it suggests that such chronic diseases do not in themselves impact negatively on a person’s mental health, although neither do they improve it. However, health status (high consumption of medication and the need for daily analgesia) does influence the level of positive mental health. This suggests that the key aspect is not the type of chronic health disease but, rather, the way in which the problem develops and the therapeutic approach it requires. As such, it is vital to develop strategies for maximizing the care and self-care of health (both physical and mental) so that people with chronic health diseases can maintain an optimal mental and physical status. In this regard, there is a need for longitudinal studies with larger samples in order to explore further the extent to which positive mental health is determined by the development of a physical health disease.

## Competing interests

The authors declare that they have no competing interests.

## Authors’ contributions

TLL, MP, JR and CF designed the study and methodology. ES picked up the sample. PMH-Research Group provided support in the conceptual process. All authors read and approved the final manuscript.

## Authors’ information

Positive Mental Health Research Group (PMH-RG) is comprised of the following members: Albacar N, Broncano M, Falcó A, Gelabert S, Lleixà MM, Mantas S, Miguel MD, Pulpón A, Sanromà M, Sequeira C, Solà M.

## Pre-publication history

The pre-publication history for this paper can be accessed here:

http://www.biomedcentral.com/1471-2458/13/928/prepub
